# Peroral cholangioscopy for detecting residual stones missed by cholangiography: Systematic review and meta-analysis

**DOI:** 10.1055/a-2676-4062

**Published:** 2025-08-26

**Authors:** Marcelo Klotz Dall'Agnol, Mateus Bond Boghossian, André Orsini Ardengh, Ygor Rocha Fernandes, Matheus de Oliveira Veras, Evellin Souza Valentim dos Santos, Tomazo Antonio Prince Franzini, Wanderley Marques Bernardo, Eduardo Guimarães Hourneaux de Moura

**Affiliations:** 1 117265 Gastrointestinal Endoscopy Unit, Department of Gastroenterology, Hospital das Clínicas da Faculdade de Medicina da Universidade de São Paulo, São Paulo, Brazil; 2 28133 Thoracic Surgery Department, University of São Paulo, São Paulo, Brazil

**Keywords:** Pancreatobiliary (ERCP/PTCD), Stones, Cholangioscopy, Diagnostic ERC

## Abstract

**Background and study aims:**

Residual bile duct stones may persist despite negative cholangiographic findings after endoscopic retrograde cholangiopancreatography, increasing risk of recurrence and complications. This systematic review and meta-analysis aimed to determine the detection rate of residual stones identified by peroral cholangioscopy (POC), alongside stone characteristics and baseline patient features.

**Methods:**

A comprehensive search was conducted in MEDLINE, Cochrane Library, EMBASE, and LILACS through August 2024. Eligible studies included patients undergoing POC after negative occlusion cholangiography. The primary outcome was the pooled residual stone detection rate. Secondary outcomes included residual stone characteristics, adverse events (AEs), and baseline clinical parameters. Subgroup analysis was performed according to cholangioscopy technique used.

**Results:**

Nine studies comprising 485 procedures were included. The pooled residual stone detection rate was 27% (95% confidence interval 23%-31%), with higher detection using digital single-operator cholangioscopy (32%) compared with direct peroral cholangioscopy (25%) and Mother-Baby systems (24%). Residual stones had a mean size of 4.51 mm, with an average of 1.55 stones per positive procedure. Mild AEs occurred in 3% of cases, with no serious complications reported. Baseline characteristics showed an average initial stone size of 12.89 mm, a mean common bile duct diameter of 15.28 mm, and lithotripsy use in 57% of cases.

**Conclusions:**

POC identified residual stones in over one-fourth of patients following negative cholangiography. Detection rates were highest with digital systems. The procedure demonstrated a strong safety profile and may play an important role in confirming complete ductal clearance.

## Introduction

Gallstone disease affects between 3.2% and 35% of the global population, with prevalence varying significantly depending on the region and ethnicity of the studied populations. Among Whites, the mean prevalence is reported to be around 10% to 15% 1 2 . Although 80% of individuals with cholelithiasis remain asymptomatic, about 20% experience symptoms such as biliary pain or acute cholecystitis, with an annual complication rate of 1% to 4%. Major complications arise when gallstones migrate to the common bile duct (CBD), increasing risk of developing cholangitis or acute pancreatitis. Given that 5% to 15% of all cholecystectomies performed in the United States involve concomitant CBD stones 2 3 , early recognition and diagnosis are crucial to prevent such complications.

Endoscopic retrograde cholangiopancreatography (ERCP), now used primarily for therapeutic purposes, is the most used procedure for treating CBD stones. However, small fragments or stones may be missed during conventional ERCP, making direct visualization devices useful for confirming CBD clearance. First described in the 1970s with the "mother-daughter" or “mother-baby” system, direct visualization of the biliary tree became possible 4 5 . Around the same time, direct peroral cholangioscopy (DPOC) was also introduced, using a forward-viewing scope that provided superior image quality and a larger working channel, although it presented greater technical challenges. In 2015, the first digital single-operator cholangioscope (D-SOC) was launched, offering enhanced image quality, improved lighting, and new tools for lithotripsy and tissue sampling 5 6 .

The recurrence rate of CBD stones after CBD clearance by ERCP is reported to range between 4% and 24% 7 8 . When the procedure is performed on patients with a significantly dilated CBD, typically > 10 to 15 mm, contrast media may fail to reveal all filling defects 9 . This issue is particularly pronounced in the presence of severe pneumobilia, which can also hinder use of intraductal ultrasonography (IDUS) 10 . In addition, when multiple or large stones (> 10 mm) are present, or lithotripsy is performed, small fragments may remain undetected 7 11 . Therefore, we conducted this systematic review and meta-analysis to assess the rate of CBD stones missed by cholangiography that were subsequently detected using POC. Subgroup analyses were performed according to the cholangioscopic technique employed. In addition, baseline patient characteristics, residual stone features, and procedure-related adverse events (AEs) were also evaluated.

### Methods

This systematic review and meta-analysis were conducted in accordance with the Preferred Reporting Items for Systematic Reviews and Meta-Analyses (PRISMA) guidelines 12 and registered in PROSPERO under the identifier CRD42024574944.

### Eligibility criteria

The included studies met the following inclusion criteria: patients who underwent ERCP for treatment of CBD stones, with no filling defects on post-treatment balloon-occluded cholangiography, and who subsequently underwent diagnostic POC. Studies were excluded if they reported positive findings on cholangiography after biliary stone treatment and/or involved patients with altered biliary anatomy (e.g., liver transplantation, Whipple procedure, biliary-enteric anastomosis). Seven full-text articles and two conference abstracts were included in this systematic review and meta-analysis.

### Search strategy and selection process

The MEDLINE, Cochrane Library, EMBASE, and LILACS databases were systematically searched between August 11 and August 21, 2024, for articles published up to August 2024 that met the predefined inclusion criteria. The following keywords were used: “cholangioscopy” or “choledochoscopy”; “residual,” “remnant,” or “missed”; “cholangiography,” “cholangiogram,” “endoscopic retrograde cholangiopancreatography,” or “ERCP”; and “stones,” “choledocholithiasis,” and “biliary calculi,” along with their respective MeSH terms and related words. The full and detailed MEDLINE search strategy is available in **Supplementary Fig. 1** .

Any article reporting clear data on the main outcome was considered eligible. If two separate publications contained data from the same population or part of it, only the most complete and recent article was included. The database search and article review were conducted independently by two authors (MKD and MBB) to assess study quality and determine eligibility. In case of disagreement, a third reviewer (AOA) was consulted.

### Data extraction and quality assessment outcomes

Key study characteristics, including the first author, year of publication, study design, country of origin, number of participants, and baseline features, were collected independently by three reviewers (MKD, MBB, and YRF). Quality and risk of bias of the included studies were independently assessed by two reviewers (MKD and MBB) using the Quality Assessment of Diagnostic Accuracy Studies-2 (QUADAS-2) tool 13 .

### Definitions

Mother-baby cholangioscopy was defined as a dual-operator cholangioscopy, in which the "baby" cholangioscope, typically non-disposable, is inserted into the working channel of the "mother" duodenoscope to assess the biliary tree. DPOC was performed by a single endoscopist using a front-view gastroscope (usually ultraslim) to directly assess the CBD, either freehand or with the aid of additional tools, such as an overtube, guidewire, and/or intraductal balloon. D-SOC was defined as a disposable, single-operator device that attaches to the duodenoscope. This device offers digital image quality, a wider field of view, and improved tip deflection, also enabling its use by a single endoscopist.

AEs were classified as major (severe) or minor (mild) according to the American Society for Gastrointestinal Endoscopy lexicon 14 .

### Outcomes

The primary outcome was the stone miss rate on cholangiography, as detected by POC, with a subgroup analysis based on type of cholangioscopic technique used. Secondary outcomes included characterization of missed stones, specifically: 1) number of residual stones and 2) their size, as well as 3) procedure-related AEs. In addition, baseline patient characteristics were assessed, including: 4) initial stone size, 5) CBD diameter, and 6) use of lithotripsy during ERCP.

### Statistical analysis

All statistical analyses were performed using R software (The R Foundation) version 4.4.1 and RStudio (Posit Software) version 2024.04.2+764, utilizing the meta, metafor, and grid packages. A random-effects model was applied to calculate estimated results for both the primary and secondary outcomes. When not provided by the studies, standard deviation and mean values were estimated using Hozo’s formula 15 for sample sizes less than 15 and Wan’s formula 16 for sample sizes greater than 15, based on sample size and range values. The corresponding results are presented in **Supplementary Fig. 2** , **Supplementary Fig. 3** , **Supplementary Fig. 4** and **Supplementary Fig. 5.**

Heterogeneity was assessed using the I² statistic, according to Cochrane guidelines 17 . The following thresholds were used for interpretation: 0% to 40% might not be important; 30% to 60% may indicate moderate heterogeneity; 50% to 90% may suggest substantial heterogeneity; and 75% to 100% may represent considerable heterogeneity.

To enhance the robustness of our findings, sensitivity analyses were conducted in cases where major methodological differences were identified among studies. Publication bias was evaluated through Kendall’s Tau and Funnel Plot.

## Results

### Study Selection

**Fig. 1:**
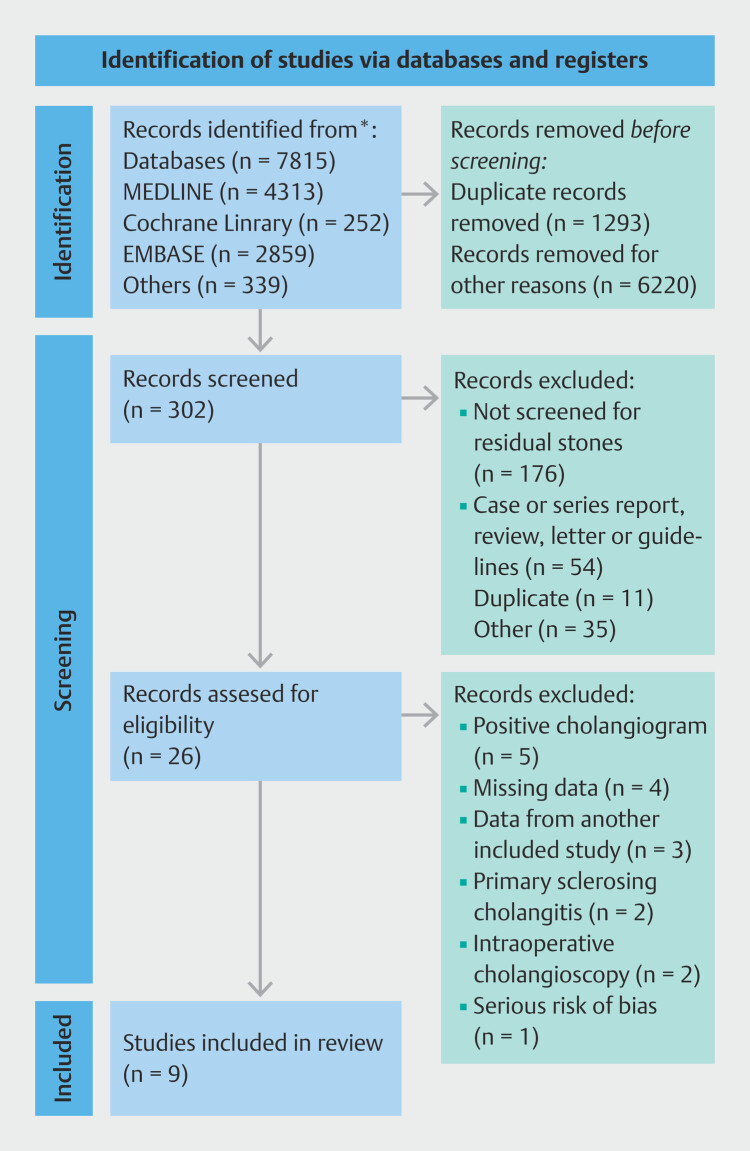
Flowchart illustrating the literature search and screening process.

After duplicate removal, 6,220 records were screened by title, followed by 302 abstracts reviewed, and 26 full-text articles assessed. After applying inclusion and exclusion criteria, nine non-randomized single-center studies (three prospective and six retrospective) were included in this systematic review and meta-analysis. The complete flowchart is presented in Fig. 1 .

### Study characteristics and quality assessment

Detailed characteristics of all included patients and studies are shown in Table 1 **.** The nine studies included in our systematic review and meta-analysis comprised 485 procedures conducted in Asia (6 studies) 8 18 19 20 21 22 , Europe (2 studies) 23 24 , and the United States (1 study) 25 . All studies employed a "tandem approach,” in which the same patient who had a negative occlusion cholangiogram during ERCP subsequently underwent diagnostic cholangioscopy to detect residual stones. Cholangioscopic technique varied with advances in endoscopic technology over time. The earliest study included in our meta-analysis 18 used mother-baby cholangioscopy, followed by five studies that used DPOC 19 20 21 22 23 . Three of the five most recent studies reported their findings using D-SOC 8 24 25 , which was first introduced in 2015. QUADAS-2 results are presented in **Supplementary Fig. 6** .

**Table:** **Table 1** Baseline characteristics of studies included in the analysis.

**First author and year of publication**	**Lee TH et al, 2022**	**Karagyozov et al, 2020**	**Sejpal et al, 2019**	**Yang et al, 2019**	**Anderloni et al, 2019**	**Omuta et al, 2015**	**Huang et al, 2013**	**Lee YN et al, 2012**	**Itoi et al, 2010**
Study design	Retrospective	Retrospective	Prospective	Retrospective	Retrospective	Retrospective	Prospective	Prospective	Retrospective
Country	South Korea	Bulgaria	USA	China	Italy	Japan	Taiwan	South Korea	Japan
Sample size (% men)	34 (NR)	38 (NR)	96 (34%)	75 (49.4%)	31 (45%)	35 (29.7%)	22 (68%)	46 (37%)	108 (60.2%)
Mean Age (range)*	72 ± 11.74 (36–87)	NR	65.1 ± 1.7 (NR)	63.3 ± 10.5 (52–79)	72.4 ± 11.6 (42–89)	81.4 (NR)	73.4 ± 11.8 (40–89)	67.5 ± 11 (NR)	73 ± 10.67 (27–91)
Technique	D-SOC	D-SOC	D-SOC	DPOC	DPOC	DPOC	DPOC	DPOC	Mother-daughter
Inclusion criteria†	^†^	At least 1: ML performed; over 3 stones; CBD > 15 mm²	At least 1: CBD > 12 mm; Lithotripsy ^†^	Choledocholithiasis treated with ERCP ^†^	All of the following: CBD > 12 mm, stone > 10 mm, dilation assisted ECRP²	^†^	^†^	All of the following: stone > 12 mm, Lithotripsy, CBD > 10 mm²	^†^
Exclusion criteria‡	Altered anatomy, uncontrolled coagulopathy, long-term biliary stents ^‡^	^‡^	B-II, Roux-en-Y, Whipple procedure ^‡^	Cholecystolithiasis, CBD < 10 mm, complete removal of single stones, combination of severe systemic diseases ^‡^	Pregnant, critically ill, cholecystitis, DAPT ^‡^	^‡^	Pregnant, critically ill, cholecystolithiasis, CBD < 10 mm³	Intrahepatic stones, bleeding tendencies, strictures, altered anatomy³	Critically ill, strictures ^‡^
CBD diameter (range), mm*	13.5 ± 1.71 (11–21)	NR	14.5 ± 0.3 (11–30)	> 10 (NR)	15.8 ± 3.7 (12–30)	14 (NR)	17.9 ± 5.1 (10–30)	18.4 ± 3.08 (10.8–31.7)	12.1 ± 1.73 (6–24)
Initial stone size (range), mm*	12.5 ± 1.37 (11–19)	NR	7.6 ± 0.4 (NR)	12.7 ± 4.2 (6–15)	12.9 ± 3.4 (10–20)	NR	13.4 ± 5.6 (5 – 25.4)	16.7 ± 2.89 (12.1–31.7)	14.6 ± 3.95 (4–45)
Prior lithotripsy (%)	ML (100%)	ML (NR)	EHL and ML (13% combined)	ML (NR)	ML (NR)	ML (24.3%)	ML (9%)	ML (100%)	EHL and ML (44% combined)
Sphincteroplasty	ES (76.4%) ES + EPBD (20.6%)	NR	ES (39%) EPDB (17%) Prior ES or EPDB (34%)	ES (29%) ES + EPBD (71%)	EPDB (83%) ES + EPBD (17%)	NR	ES (5%) EPBD (68%) ES + EPBD (27%)	ES (78.3%) ES + EPBD (21.7%)	ES (91.7%) Prior ES (8.3%)
Clearence rate	90.9%	NR	NR	NR	100%	100%	100%	84.6%	100%
Symptoms during follow-up	NR	NR	NR	NR	NR	NR	4 recurrent stones (2 with and 2 without residual stones)	NR	NR
*All baseline characteristics were reported by the original studies as means with standard deviations. Omuta et al. (2015) did not provide the standard deviation for age and CBD diameter. ^†^ All studies reported negative cholangiography findings. ^‡^ All studies excluded patients with filling defects observed on cholangiography. B-II, Billroth II; CBD, common bile duct; DAPT, dual antiplatelet therapy; DPOC, direct peroral cholangioscopy; D-SOC, digital single-operator cholangioscopy; EHL, electrohydraulic lithotripsy; EPBD: endoscopic papillary balloon dilatation; ERCP, endoscopic retrograde cholangiopancreatography; ES, endoscopic sphincterotomy; ML, mechanical lithotripsy; NR, not reported.									

### Results of stone miss sate

**Fig. 2:**
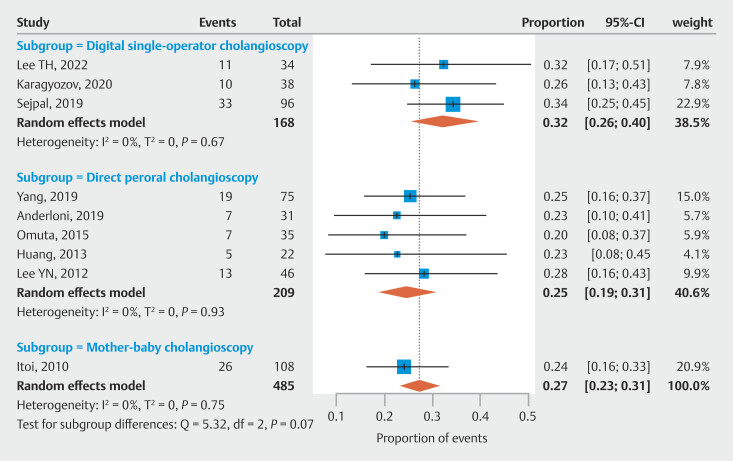
Proportion of residual stones missed by cholangiography and detected by tandem cholangioscopy.

As the primary outcome, the stone miss rate for balloon-occluded cholangiography across all nine studies included in our meta-analysis was estimated at 27% (95% confidence interval [CI] 0.23–0.31, N = 9, I² = 0%), with a higher detection rate observed using D-SOC (32%; 95% CI 0.26–0.40; N = 3, I² = 0) compared with DPOC (25%; 95% CI 0.19–0.31; N = 5, I² = 0) and mother-baby cholangioscopy (24%; 95% CI 0.16–0.33) in subgroup analysis. The forest plot is shown in Fig. 2 .

### Results of residual stones

**Fig. 3:**
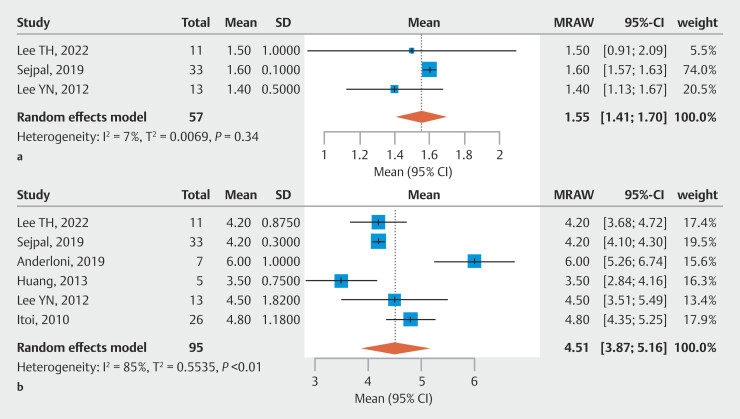
**a** Mean number of residual stones detected via cholangioscopy. **b** Average size (mm) of residual stones detected via cholangioscopy.

In a three-article analysis presented in Fig. 3 **a** , the mean number of stones was 1.55 (95% CI 1.41–1.70, N = 3, I² = 7%). The average size of the residual stones was 4.51 mm (95% CI 3.87–5.16, N = 6, I² = 85%; Fig. 3 **b** ).

### Results of adverse events

**Fig. 4:**
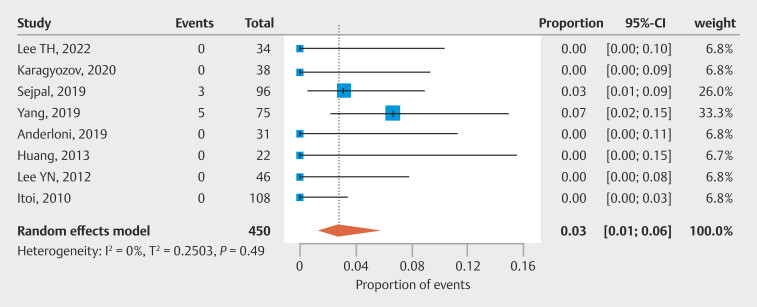
Mild adverse events associated with the tandem approach of cholangiography and cholangioscopy.

No serious AEs were reported across the 485 procedures included in our meta-analysis. Mild AEs (such as mild cholangitis and mild or moderate pancreatitis) occurred in 3% of cases (95% CI 0.01–0.06, N = 8, I² = 0%; Fig. 4 ).

### Results of mean baseline characteristics

**Fig. 5:**
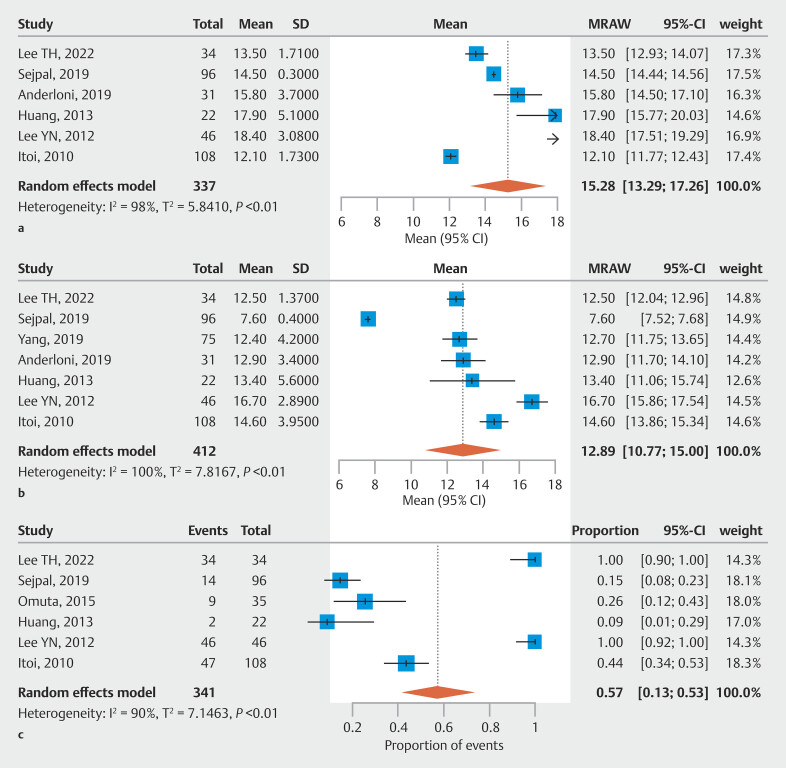
Baseline characteristics. **a** Mean common bile duct diameter in patients undergoing both cholangiography and cholangioscopy. **b** Mean size of the initial stone (mm) in patients undergoing both cholangiography and cholangioscopy. **c** Proportion of patients who underwent lithotripsy during ERCP.

To explore baseline patient characteristics associated with the indication for cholangioscopy in detection of residual stones, we analyzed CBD diameter, initial stone size, and proportion of patients who underwent lithotripsy prior to a negative cholangiography. Among the population in our meta-analysis (both with and without residual stones), mean CBD diameter was 15.28 mm (95% CI 13.29–17.26, N = 6, I² = 98%; Fig. 5 **a** ). The average size of the initial stone during ERCP was 12.89 mm (95% CI 10.77–15.00, N = 7, I² = 100%; Fig. 5 **b** ). For lithotripsy — both mechanical and/or electrohydraulic — the proportion of patients who underwent lithotripsy during ERCP was 57% (95% CI 0.13–0.93, N = 6, I² = 90%; Fig. 5 **c** ).

### Publication bias and sensitivity analysis

Sensitivity analysis was conducted only in cases in which major methodological differences were identified among studies. This analysis was conducted for both the primary and secondary outcomes, demonstrating that excluding outliers improved meta-analysis results for the number of residual stones and mild AEs. Forest plots are available in **Supplementary Fig. 7** and **Supplementary Fig. 8** . To assess publication bias, Kendall’s Tau was 0 ( *P* = 1.0), indicating a low risk of publication bias. The funnel plot is presented in **Supplementary Fig. 9** .

## Discussion

For decades, balloon-occluded cholangiography was considered the gold standard for assessing choledocholithiasis and confirming CBD clearance 26 27 . However, after introducing direct visualization techniques, our study revealed a 27% rate of missed stones during ERCP, which were detected using POC in a tandem approach. This represents the first systematic review and meta-analysis to address this issue.

As previously mentioned, up to 24% of patients can develop recurrent CBD stones within 3 years following successful stone retrieval via ERCP 28 29 . Cheon et al. 7 hypothesized that, while development of new primary stones in the biliary ducts is possible, a portion of this recurrence rate may be attributed to residual stones not visible on occlusion cholangiograms, which act as nidi for formation of larger stones 30 . Primary risk factors associated with stone recurrence after ERCP are described as a large CBD (> 12 mm), large stones (> 10 mm), lithotripsy, multiple stones, stent implantation, periampullary diverticulum, and bile duct strictures 11 28 29 , with at least one of the first three commonly being used as inclusion criteria in the majority of the studies included in our analysis.

As highlighted above, CBD diameter is one of the most significant risk factors for stone recurrence 31 32 . When the diameter exceeds 15 to 18 mm, achieving a high-quality occlusion cholangiogram and complete bile duct clearance becomes more challenging. This occurs because extraction balloons typically available are often no larger than 18 mm 18 . As a result, small residual stones or fragments may be missed, potentially leading to persistent symptoms and a higher risk of complications, including cholangitis and pancreatitis. To better understand the clinical relevance of these missed stones, controlled studies with long-term follow-up are needed to compare outcomes between patients undergoing peroral cholangioscopy and those receiving no additional intervention after a negative occlusion cholangiogram.

In all included studies except one, cholangioscopy was performed immediately after a negative occlusion cholangiogram. The study by Itoi et al. 20 was the only one to conduct cholangioscopy as a second-look procedure, approximately 6 days after the initial ERCP. This study reported a higher mean number of residual stones, raising the possibility of interval migration from the gallbladder to the CBD. Due to this methodological discrepancy—and its classification as an outlier in sensitivity analyses—it was excluded from the pooled analysis of residual stone counts ( **Supplementary Fig. 7** ).

Due to insufficient data, we were unable to reliably assess potential risk factors through meta-regression. Although meta-regression was attempted, none of the baseline characteristics showed statistical significance. This likely reflects the homogeneity of the included populations, which predominantly comprised patients with markedly dilated bile ducts and large initial stones. As a result, meaningful comparisons with patients presenting with smaller CBD diameters or stone sizes were not feasible. To ensure methodological transparency, full meta-regression results are provided in meta-regression results in **Supplementary Fig. 10** , **Supplementary Fig. 11** , **Supplementary Fig. 12** , and **Supplementary Fig. 13** .

POC remains a relatively high-cost procedure 6 32 . Nonetheless, previous studies have suggested that its use in management of difficult biliary stones 33 34 and in evaluation of indeterminate biliary strictures 33 35 may be cost-effective, particularly when performed early in the disease course. Although formal cost analyses were not conducted in the studies included in our systematic review, it is plausible that the combined costs of repeat ERCPs, potential emergency department visits, and prolonged hospitalizations may exceed those of a single tandem cholangioscopy in appropriately selected cases.

For example, the clearance rate for residual stones detected by cholangioscopy was reported in six of the nine included studies. Four studies demonstrated a 100% clearance rate, whereas the remaining two reported rates of 84.6% and 90.9%, respectively. In all cases, clearance was confirmed by direct cholangioscopic visualization. This high success rate may reduce need for subsequent procedures, thereby minimizing downstream healthcare costs. Furthermore, given that D-SOC has only been widely available for less than a decade — and considering the continuous evolution in endoscopic technologies — it is reasonable to anticipate a gradual reduction in procedural costs over time.

In our analysis, cholangioscopy detected a mean of 1.55 stones per patient that were missed by balloon-occluded cholangiography, with an average size of 4.51 mm per stone. Given that most patients had undergone a large endoscopic sphincterotomy and/or endoscopic papillary balloon dilation, it is plausible that some of these small residual fragments might gradually migrate over time, potentially posing a low risk of future complications. However, only three studies included in our analysis reported follow-up. Huang et. al 20 reported that four patients experienced recurrence of CBD stones after a mean follow-up of 17.5 months. Two of these patients had residual stones previously detected by tandem cholangioscopy, whereas the other two did not. Because the authors did not provide further clinical details and the overall number of recurrences was low, it is not possible to determine whether these outcomes were associated with residual fragments or simply reflected persistent risk factors for stone formation.

When combining invasive and potentially high-cost procedures, careful patient selection becomes essential. Although the included studies were not powered to formally identify predictors of residual stones, most shared similar inclusion criteria: a CBD diameter typically greater than 10 to 12 mm, an initial stone size exceeding 10 to 12 mm, and use of lithotripsy in approximately half of the cases. Despite some heterogeneity in baseline characteristics—largely attributable to the small sample sizes of individual studies—it may be reasonable to consider POC after a negative occlusion cholangiogram when at least one, and preferably two, of these three factors are present.

Our study has several strengths. First, it is the first systematic review and meta-analysis to evaluate use of cholangioscopy in evaluation of residual stones missed by cholangiography, utilizing a large sample of 485 tandem procedures. Second, it demonstrated no heterogeneity in analysis of the primary outcome, its subgroup analysis, or AE outcome, with low heterogeneity observed in mean number of residual stones. Third, it revealed no publication bias and included a thorough quality assessment, excluding articles with confounding or unclear data.

However, our study also has several limitations. First, none of the included articles are randomized controlled trials. This is likely due to the challenges of conducting such studies in evaluation of residual stones. Second, two of the included articles were conference abstracts 21 24 . Although not peer-reviewed full-text articles, both presented findings consistent with the full-text articles, particularly regarding the primary outcome. Third, some secondary outcomes, such as size of residual stones, as well as mean baseline characteristics (CBD diameter, initial stone size, and prior lithotripsy), showed high heterogeneity. To address this, we employed a random-effects model for all results in our analysis, ensuring more robust and reliable estimates. Fourth, meta-regression was limited by the small number of studies and insufficient data, particularly for patients with smaller initial stones and CBD diameters, preventing meaningful comparisons. Nonetheless, we provided those results in **Supplementary Materials** , as highlighted above. Fifth, only the more recent studies utilized D-SOC, which has become the predominant technique in recent years. Despite this, older techniques such as DPOC and mother-baby systems still demonstrated substantial detection rates for residual stones (24%-25%), compared with 32% with D-SOC. These findings suggest that, when used in appropriately selected patients, D-SOC could offer even greater sensitivity for detecting stones missed by conventional cholangiography.

## Conclusions

In conclusion, our systematic review and meta-analysis demonstrated that POC identified residual stones in over one-quarter of cases following negative cholangiography, with improved detection rates achieved using D-SOC. Residual stones had a mean size of 4.51 mm, with an average of 1.55 stones detected per procedure, highlighting the utility of this approach in identifying missed stones. Furthermore, the procedure exhibited a favorable safety profile, with a low incidence of mild AEs and no reports of serious complications, further supporting its efficacy and safety in the detection of residual stones.

## Supplementary Material

Supplementary Material Zusatzmaterial
