# Supplementary material for: Peroral cholangioscopy for detecting residual stones missed by cholangiography: Systematic review and meta-analysis
Source: Endosc Int Open. 2025 Aug 26;13:a26764062. doi: 10.1055/a-2676-4062 (PMC12417788; doi:10.1055/a-2676-4062)
Supplement: Supplementary file 1 — Supplementary Material [file 10-1055-a-2676-4062_26793333.pdf]

Supplementary Figure 1. Detailed search strategy.

```
(cholangioscopy OR Choledochoscopy OR spyglass OR EyeMax OR DCS OR
cholangiopancreatoscopy OR pancreatoscopy OR peroralcholangioscopy OR DPOC OR
fluoroscopy-free OR SOC OR choledochofiberscopic OR POC OR cholangioscope OR
choledochoscope)
AND
(residual OR uncomplicated OR non-complex OR clearance OR retained OR retrieval OR
defects OR missed OR incomplete OR fragments OR fragmented OR removal OR removed
OR unsuccessful OR refractory OR removal OR extraction OR balloon-occluded OR remnant
OR extraction OR failure)
AND
(Cholangiography OR Cholangiogram OR Cholangiopancreatography, Endoscopic Retrograde OR
Conventional OR ERCP OR Endoscopic Sphincterotomy OR Endoscopic Sphincterotomies OR
Endoscopic Papillotomy OR Endoscopic Biliary Sphincterotomy OR Biliary Sphincterotomies,
Endoscopic OR Endoscopic Papillotomies OR Cholangiopancreatographies, Endoscopic
Retrograde OR Fluoroscopy OR ERC)
OR
(Biliary Calculi OR Gall Stones OR Common Bile Duct Calculi OR Gallstones OR Common Bile
Duct Gall Stone OR Common Bile Duct Gallstone OR Gall Stone OR Gallstone OR Cholelithiasis
OR Choledocholithiasis OR Bile Stones OR Calculi OR Calculus OR Stone)
```

Supplementary Figure 2. Results of the standard deviation calculation for the number of residual stones.

Study	n	min_val	max_val	SD
Lee TH, 2022	11	1	5	1.0000000
Lee YN, 2012	13	1	3	0.5000000
Itoi, 2010	26	1	4	0.5883484

Supplementary Figure 3. Results of the standard deviation calculation for the size of residual stones.

Study	n	min_val	max_val	SD
Lee TH, 2022	11	2.1	5.6	0.875000
Huang, 2013	5	2.0	5.0	0.750000
Lee YN, 2012	13	2.3	9.6	1.825000
Itoi, 2010	26	2.0	8.0	1.176697

Supplementary Figure 4. Results of the standard deviation calculation for common bile duct diameter.

Study	n	min_val	max_val	SD
Lee TH, 2022	34	11.0	21.0	1.714986
Lee YN, 2012	46	10.8	31.7	3.081537
Itoi, 2010	108	6.0	24.0	1.732051

Supplementary Figure 5. Results of the standard deviation calculation for initial stone size.

Study	n	min_val	max_val	SD
Lee TH, 2022	34	11.0	19.0	1.371989
Lee YN, 2012	46	12.1	31.7	2.889862
Itoi, 2010	108	4.0	45.0	3.945227

Supplementary Figure 6. Quality Assessment of Diagnostic Accuracy Studies-2 (QUADAS-2) checklist of included studies.

QUADAS-2 Results[1,2,3]

Risk of Bias and Applicability Concerns

	Risk of Bias				Applicability Concerns		
Study	Patient Selection	Index Test	Reference Standard	Flow and Timing	Patient Selection	Index Test	Reference Standard
Lee TH, 2022	🟢	🟢	🔵	🟢	🟢	🟢	🟢
Karagyozov, 2020	🟢	🟢	🔵	🟢	🟢	🟢	🟢
Yang, 2019	🟢	🟢	🔵	🟢	🟢	🟢	🟢
Sejpal, 2019	🟢	🟢	🔵	🟢	🟢	🟢	🟢
Anderloni, 2019	🟢	🟢	🔵	🟢	🟢	🟢	🟢
Omuta, 2015	🟢	🟢	🔵	🟢	🟢	🟢	🟢
Huang, 2013	🟢	🟢	🔵	🟢	🟢	🟢	🟢
Lee YN, 2012	🟢	🟢	🔵	🟢	🟢	🟢	🟢
Itoi, 2010	🟢	🟢	🔵	🔴	🟢	🟢	🟢

[1] 🟢: Low risk
[2] 🔵: Unclear risk
[3] 🔴: High risk

Supplementary Figure 7. Sensitivity analysis for the number of residual stones outcome.

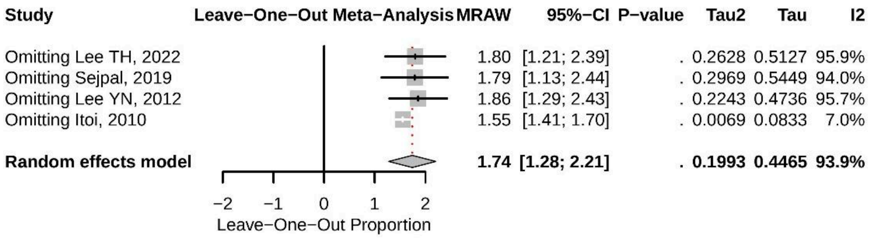

Supplementary Figure 8. Sensitivity analysis for the adverse events outcome.

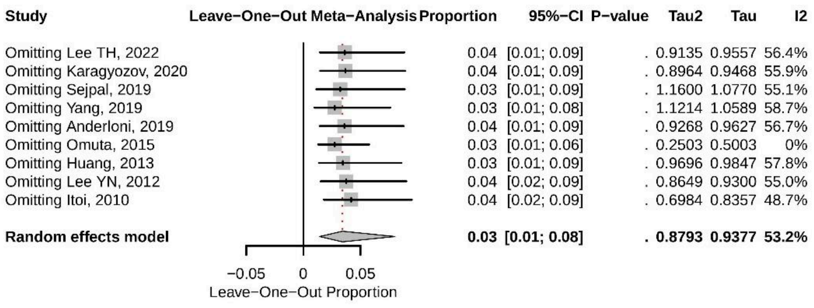

Supplementary Figure 9. Funnel plot assessing publication bias.

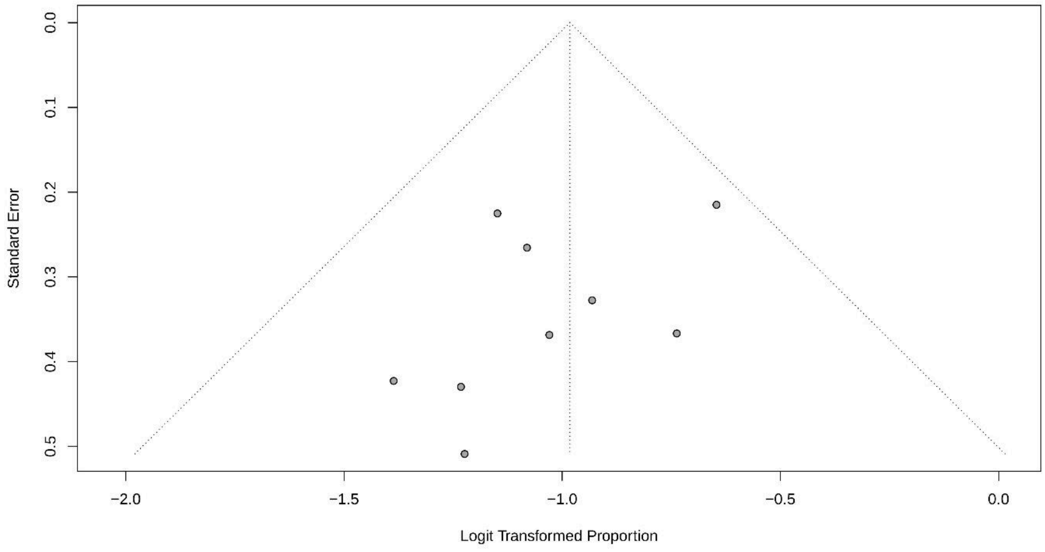

Supplementary Figure 10. Meta-regression results for CBD diameter.

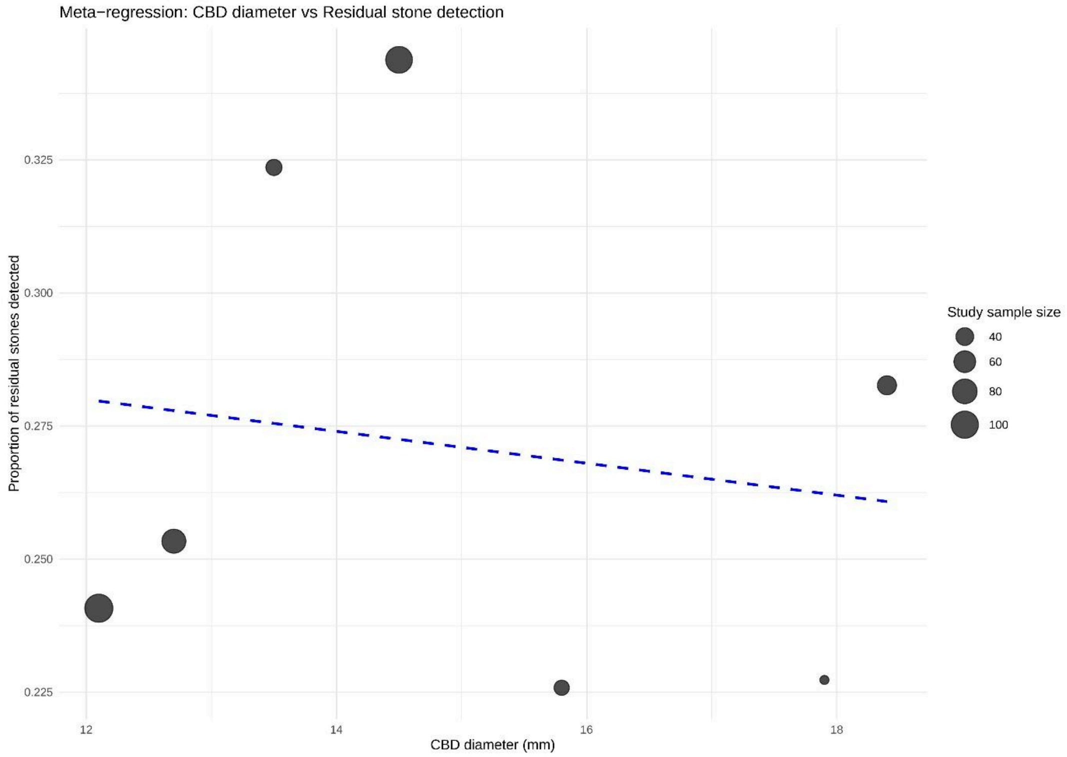

Supplementary Figure 11. Meta-regression results for initial stone size.

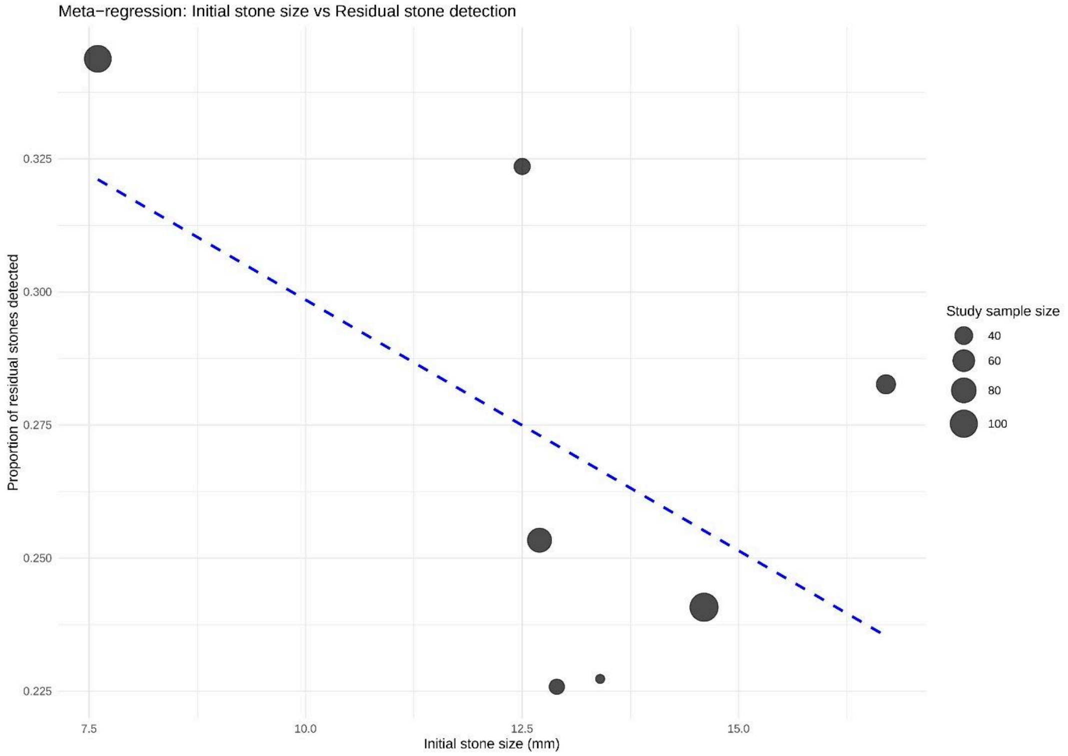

Supplementary Figure 12. Meta-regression results for prior lithotripsy.

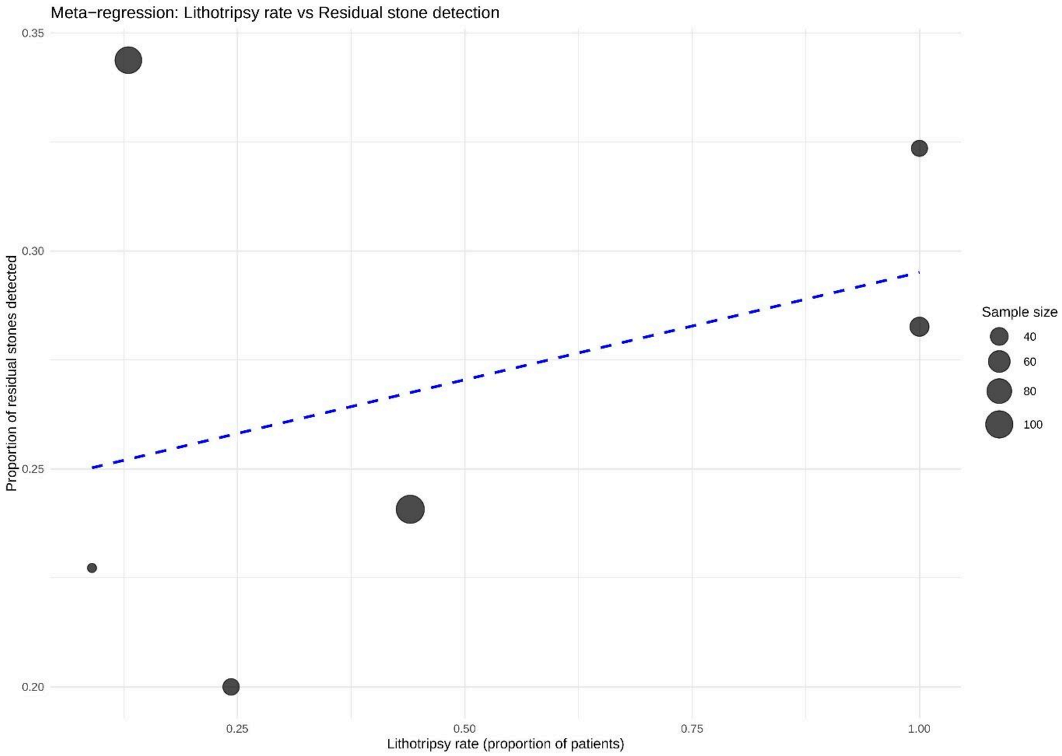

Supplementary Figure 13. Summary table of meta-regression analyses for CBD diameter, initial stone size, and lithotripsy rate.

Moderator	Pooled OR	95% CI (OR)	p-value
CBD diameter (mm)	1.02	0.91 to 1.13	0.778
Initial stone size (mm)	0.95	0.88 to 1.02	0.134
Lithotripsy rate (per 100%)	1.05	0.47 to 2.35	0.896
